# Hand Asymmetry Does Not Relate to Key Life History Traits in Post-Menopausal Contemporary Finnish Women

**DOI:** 10.1371/journal.pone.0034661

**Published:** 2012-04-06

**Authors:** Stefan Van Dongen, Ellen Sprengers, Samuli Helle

**Affiliations:** 1 Evolutionary Ecology Group, Biology Department, Antwerp University, Antwerp, Belgium; 2 Section of Ecology, Department of Biology, University of Turku, Turku, Finland; University of Jyväskylä, Finland

## Abstract

Associations between fluctuating asymmetry (FA, a putative marker of developmental instability, DI) and life history traits have received a great deal of attention in the non-human literature. However, the patterns found are very heterogeneous and generalizations are difficult to make. In humans, only a few studies have related FA to life histories and fitness. In this paper we study such relationships using hand FA and several key life history traits in 209 post-menopausal Finnish women born between 1946 and 1958. Asymmetry measurements were based on scans of the hands and the life histories of these women were collected using questionnaires. No significant associations were detected and trends were opposite to expectations. We did find evidence for directional asymmetry, as traits in the right hand were larger on average. This may be due to handedness, questioning the usefulness of hand FA as a measure of DI. We conclude that future studies in humans should carefully examine the usefulness of traits as measures of DI.

## Introduction

Fluctuating asymmetry (i.e., FA, small random deviations from perfect bilateral symmetry), is assumed to result from developmental instability (i.e., DI, the inability of an individual to buffer its development against directionally random perturbations) [1]. Associations between FA and various components of fitness or measures of stress have led to the conclusion that DI could reflect individual genetic quality and may play an important role in sexual and natural selection [1]. However, the literature is very heterogeneous and we understand little about the nature of this variation among studies [1]. A recent review of the literature in human FA, for example, showed that indeed, on average, there is a modest increase in DI with various health problems (e.g., congenital abnormalities, schizophrenia, …), and decrease of it with attractiveness and aspects of sexual behavior (e.g., age of first sexual contact, number of sexual partners, …) with an average effect size of about 0.3 [2]. Yet, effect sizes greatly varied among studies and this variation could not be explained by the type of trait or type of stress, health or quality measure [2]. Clearly, there is a need for further research attempting to understand this heterogeneity and the importance of DI in human evolutionary biology.

In general, there is accumulating evidence that variation in life histories in modern humans is not static, but rather is currently evolving and under directional selection [3]. Most general life history traits under current selection are the higher number of offspring born, the earlier age at the start and at the later end of reproduction and, thus, younger age at menarche and older age at menopause [4], [5]. Better environmental conditions and/or better locally adapted genes lead to earlier start of reproduction, longer reproductive tenure and higher offspring number. Since FA has been put forward as such a possible measure of ‘quality of development’ (either environmentally or genetically induced), associations between FA and life history traits can be predicted in humans. In this paper we test if individuals that ‘enjoyed’ more optimal conditions/‘better’ genes during their early development (i.e., are more symmetric having low FA) would differ in their life history later in life from individuals that experienced suboptimal development (i.e. are more asymmetric having high FA).

A few earlier studies in humans have reported associations between FA and life history traits. Møller et al. [6] found negative associations between breast FA and the number of children and positive associations between breast FA and age at first reproduction in two populations. This suggests that more symmetric (‘high quality’) women have more children and start reproducing earlier, which indicates higher fitness [4], [7]. These results were repeated by Manning et al [8] in a larger sample. If FA reflects a genome-wide effect of developmental instability and genetic quality, one would predict to find similar results for other trait as wells. However, stronger effects may occur for traits like breast FA because sexually selected traits may show stronger associations with life history traits than non-sexually selected traits like hands [9]. Positive associations between body mass index and bodily FA in women suggested a role for BMI in sexual selection, where higher BMI is rated/experienced as less attractive [10], [11]. Poorer ecological conditions may also lead to lower offspring survival and/or selective relative investment in male and female offspring. For example, higher BMI is found to be associated with the excess of male births [12] and access to resources is likely to aid offspring survival [13]. Therefore, offspring survival and sex ratio will also be included in our attempt to provide a broad assessment of life history characteristics.

The study of FA is characterized by potential problems and biases. Levels of FA are usually small in the order of 1% relative to trait size. Therefore, FA can easily get confounded with measurement error (ME) and other forms of asymmetry [1]. The use of FA as a measure of DI implicitly assumes that both sides develop under exactly the same conditions. However, especially in humans, behavioral lateralization (i.e., handedness) after birth may result in different mechanical loads especially in limbs, which in turn may affect development [14], [15], and may not reliably reflect DI. Perhaps unfortunately, asymmetry in adult limbs is often used in asymmetry studies, and it is therefore crucial to test for directional asymmetry (DA). Traits showing DA can then be excluded or FA measurements can be corrected for possible presence of DA.

In this study, we relate hand FA to key life history traits in contemporary post-menopausal women born between 1946 and 1958 in Finland. In contrast to previous studies, women included in this study had ended their reproductive career so that the LRS could be defined (given that there was minimal offspring mortality. Furthermore, we control for possible confounding factors such as education level (an important determinant of life history traits like family size and age of reproduction) and birth cohort and area to account for possible temporal and geographic variation. We analyze multivariate associations between hand asymmetry and the following life history traits: body mass index (BMI), lifetime number of children born, age of first and last reproduction, age at menarche, proportion of offspring surviving until 18 years old and proportion of male offspring at birth (secondary sex ratio). As such it should provide an overall assessment of the relationship between FA and life history strategy among the post-menopausal women studied.

## Materials and Methods

### Study population

Complete lifetime reproductive history using questionnaires and scans (Canon Canoscan D66OU) of the right and left hand of 209 women born between 1946–1958 in Finland were collected during 2006 [16]. These women consist presumably a random sample of ca. 50-, 55- and 60-year-old voluntaries that participated in the Finnish national screening program of cervical cancer in 2006. The relatedness of these women is unknown, but it must be negligible as these women represent a geographically diverse sample from Finland [17]. From the 1960 s onwards, when the earliest cohort of the women studied started to reach sexual maturity, Finnish women generally made use of modern contraceptive methods (78% of the women included in this study reported that they have used contraceptives during their lifetime). Life-history variables recorded for the purposes of the current study were body mass index (BMI), number of children born during lifetime, age of first and last reproduction, age at menarche, the proportion of offspring surviving until 18 years old and the proportion of male offspring (i.e. secondary sex ratio). We measured lifetime reproductive success by two factors, i.e., the total number of offspring born during lifetime and their probability of survival to age 18. We also recorded women's birth area (classified to be born in North-, South- or West-Finland or born abroad), birth cohort (1946–47, 1951–52 or 1956–58) and their education (elementary school, secondary school or university degree), which is an important determinant of, for example, family size in modern populations [18].

### Measurement of FA

**Figure 1:**
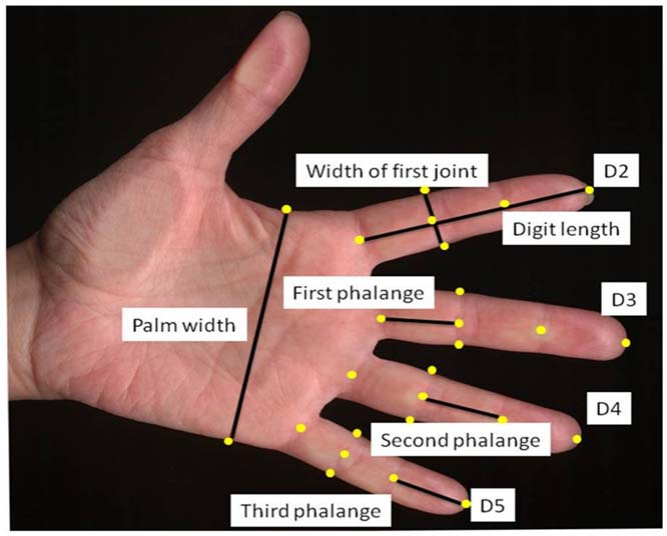
Overview of measurements performed on scans of post-menopausal women. On each scan 26 landmarks (yellow dots) were placed using ImageJ. On the basis of those coordinates, the width of the palm of the hand was calculated. For digits 2 to 5 (D2, D3, D4 and D5), the total digit length, the length of each phalange and the width of the first joint was calculated.

Scans were analyzed using ImageJ (ver. 1.42 freely available at http://rsbweb.nih.gov/ij/) [19]. One of us (ES) digitized a total of 26 landmarks on each scan (Fig. 1). These landmarks were selected such that they reflect different aspects of the hand. The lengths of different digits or fingers are typically measured from the bottom crease where the finger joins the hand to the tip of the finger [15], [16], [17]. Therefore, these landmarks were included for digits 2, 3, 4 and 5 (Fig. 1). In addition, the length of each of the three phalanges could be measured and analyzed by adding two landmarks on the crease of the joints of the phalanges for each digit (Fig. 1). The width of the first joint of digits 2, 3, 4 and 5 was measured by placing a landmark on both sides of the joint where the so-called PIP crease reached the outer lines of each digit (Fig. 1). Finally, the width of the palm of the hand was measured from the tip of the distal palmar crease to the tip of the proximal palmar crease (Fig. 1) [15]. In a second independent session, all landmarks were digitized again to allow determining measurement error due to the placing of landmarks on the individual scans. Since we did not have repeated scans, we underestimated the overall rate of measurement error. However, Van Dongen et al. [15] found low error due to repeated scanning of hands, so we do not expect this to be problematic. Based on the coordinates, a total of 21 Euclidian distances were calculated (Fig. 1).

For each distance we determined the amount of real FA, the degree of measurement error and tested for directional asymmetry using a mixed regression model, with side as continuous covariate (left = −0.5 and right = 0.5) and individual and side-by-individual interaction as random effects [1]. Signed FA values were obtained from the random slopes of the mixed regression model and were thus corrected for potential directional asymmetry. The hypothetical repeatability, a measure of how closely the FA measures reflect variation in the underlying DI, was obtained following Whitlock (1998) [20]. Between-trait correlations in signed FA were also tested to explore developmental integration. The underlying idea is that traits which share a developmental pathway (i.e., developmentally integrated traits) also share the developmental errors and should show correlations in the signed FA [21]. Understanding developmental integration is relevant here as it allows to anticipate if different measurements on the hand provide independent information about DI [15]. Hand asymmetry (hand FA) was calculated as the average asymmetry across all traits after standardization of FA of each trait. An analysis was done for all traits, and for all traits not showing DA and thus not/less affected by handedness.

### Statistical analyses

Associations between hand FA and life history traits were analyzed using a multivariate linear regression model. Body mass index, number of children, age of first and last reproduction, age at menarche, offspring survival until 18 years old and the proportion of male offspring were treated as response variables following a multivariate normal distribution. Hand FA (all traits or traits without DA) was used as predictor. We controlled for educational level, birth cohort and birth area in these analyses. Overall multivariate tests as well as parameter estimates and tests for each life history trait separately were provided. Strongest associations (significant or not) were presented graphically.

## Results

**Figure 2:**
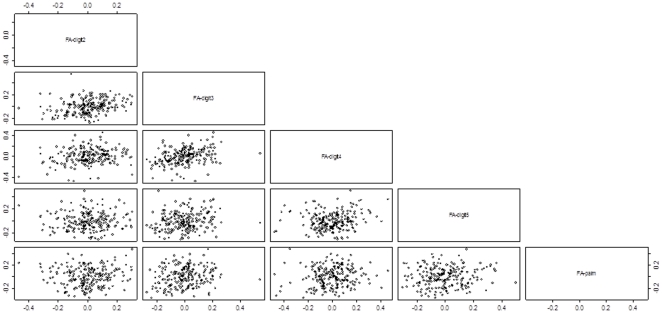
Associations in the signed asymmetries between digit lengths and hand palm width.

Table 1 provides the descriptive statistics of the asymmetry measurements in all traits. Overall, ME was relatively low. Out of 21 traits, 13 showed significant directional asymmetry, where in 11 cases, the right side was larger compared to the left. On average, the measurements on the right side were 0.8% larger. Asymmetry values were corrected for this presumed directional asymmetry. The hypothetical repeatability R was positive in several traits and equaled on average 0.07, suggesting that the observed asymmetries in hands reveal a small amount of variation in DI only. However, combining data from different traits does not provide independent information of individual DI since there appear to be correlations in the signed FA values suggesting that hands develop as an integrated complex trait. Table 2 provides these correlations for digit lengths and palm width. Adjacent digits showed stronger developmental integration (Table 2, Fig. 2). This was also the case for the phalanges and widths of the first joint (data not shown). Table 3 provides the descriptive statistics of the key life history traits included in this study.

**Table 1:** Descriptive statistics of asymmetry measures.

Trait	real FA	ME	DA	R-L (% of size)	R
Palm width:					
P	0.031	0.002	χ^2^_1_ = 40.5^***^	**0.08 (1.0)**	−0.11
Digit lengths:					
D2	0.019	0.002	χ^2^_1_ = 61.0^***^	**0.08 (1.1)**	0.12
D3	0.016	0.001	χ^2^_1_ = 24.1^***^	**0.05 (0.6)**	0.08
D4	0.024	0.003	χ^2^_1_ = 6.08^*^	**0.03 (0.4)**	0.14
D5	0.022	0.002	χ^2^_1_ = 3.90	−0.02 (−0.3)	0.03
Lengths of first phalange of digits:					
D21	0.015	0.003	χ^2^_1_ = 4.40^*^	**−0.02 (−0.8)**	0.10
D31	0.013	0.003	χ^2^_1_ = 5.32^*^	**0.02 (0.8)**	0.05
D41	0.024	0.004	χ^2^_1_ = 1.18	0.01 (0.1)	0.20
D51	0.017	0.003	χ^2^_1_ = 2.74	0.02 (0.2)	0.16
Lengths of second phalange of digits:					
D22	0.011	0.002	χ^2^_1_ = 10.5^***^	**0.03 (1.2)**	0.16
D32	0.009	0.003	χ^2^_1_ = 1.08	0.01 (0.3)	0.09
D42	0.015	0.003	χ^2^_1_ = 0.35	0.00 (0.1)	0.17
D52	0.011	0.003	χ^2^_1_ = 19.4^***^	**−0.04 (2.2)**	0.15
Lengths of third phalange of digits:					
D23	0.008	0.001	χ^2^_1_ = 114^***^	**0.08 (2.9)**	0.03
D33	0.008	0.001	χ^2^_1_ = 5.58^*^	**0.02 (0.7)**	0.19
D43	0.009	0.001	χ^2^_1_ = 2.24	0.01 (0.4)	0.15
D53	0.009	0.002	χ^2^_1_ = 0.15	0.00 (0.0)	0.00
Width of first joint of digits:					
W2	0.006	0.001	χ^2^_1_ = 14.4^***^	**0.02 (1.1)**	0.28
W3	0.003	0.001	χ^2^_1_ = 72.3^***^	**0.04 (2.1)**	−0.15
W4	0.003	0.001	χ^2^_1_ = 22.8^*^	**0.02 (2.2)**	0.00
W5	0.005	0.001	χ^2^_1_ = 2.37	0.01 (0.4)	0.11

We measured the total length of digits 2–5. We also measured the lengths of phalanges 1–3 in these digits as well as the width of the first joint in digits 2–5 (see also figure 1). Real fluctuating asymmetry (real FA) and measurement error (ME), a test of directional asymmetry (DA) and a measure of this difference (right-left both in absolute terms and as a proportion of trait size) as well as the hypothetical repeatability (R) are given.

**Table 2:** Pearsons correlations in signed asymmetries of digit lengths and hand palm width.

	D2	D3	D4	D5
D3	**0.27**			
D4	**0.18**	**0.32**		
D5	0.03	**0.19**	**0.16**	
Palm	0.07	**0.18**	0.10	0.09

Significant correlations (p<0.05) are indicated in bold.

**Table 3:** Descriptive statistics of the key life history traits studied here.

	Mean (SD)	N	minimum	maximum
BMI	26.4 (4.6)	201	18.2	48.4
Age of first reproduction	24.3 (4.8)	186	16	43
Number of offspring	1.9 (1.0)	209	0	5
Age of last reproduction	29.1 (5.1)	186	17	43
Age at menarche	13.0 (1.5)	206	10	19
Proportion of sons	0.54 (0.39)	190	0	1
Offspring survival	0.98 (0.10)	186	0.25	1.33

**Figure 3:**
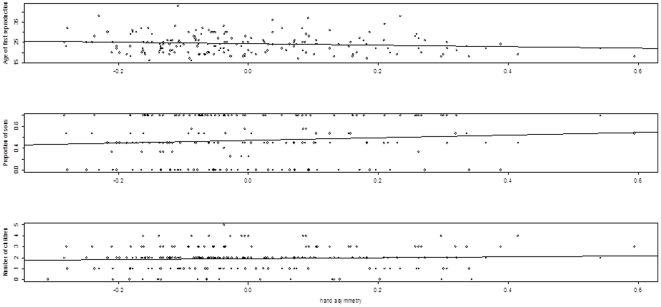
Association between hand asymmetry (all traits) and age of first reproduction (top), offspring sex ratio (middle) and number of children (bottom) in post-menopausal Finnish women. None of the associations was statistically significant, but these associations showed the highest effect sizes (Table 2). The sign of the slopes were opposite to what was found in previous studies (see text).

Table 4 provides significance tests for the average asymmetry (all traits and traits without significant DA only) in relation to the different life history traits studied, correcting for birth place, birth cohort and educational level. Multivariate tests showed no association between hand FA and female life history (Table 4), but individual parameter estimate for age at first reproduction when considering all traits was just statistically significant (yet not after correction for multiple testing). Strongest associations were found for age at first reproduction, the proportion of sons and the number of offspring born (Table 4). However, these associations were opposite to patterns observed in other studies (Fig. 3; Table 4). To account for possible non-linear associations with BMI (as intermediate values may be optimal), we also added BMI^2^ to the models, yet, leading to the same conclusions (details not shown). As expected, there was a significant overall effect of education level on the life history traits (F_14,195_ = 2.19, p = 0.01). Women with a university degree started and ended reproducing later and had fewer children (p<0.05). All other life history traits did not differ with education level (p>0.1). No effects of birth cohort (F_14,195_ = 0.97, p = 0.48) and birth place (F_21,195_ = 1.03, p = 0.43) were observed. An analysis removing the latter two statistically non-significant effects gave qualitatively similar results (not shown). Although statistical power was very limited, we also explored interactions between the effects of asymmetry and both birth cohort and education level, but none were close to significance (p>0.3), suggesting that associations did not vary with cohort or education.

**Table 4:** Overview of significance tests for associations between individual life history traits and hand asymmetry.

Factor	All traits	traits without DA
BMI	β = 0.14 (t_195_ = 0.60, p = 0.55)	β = 0.05 (t_195_ = 0.32, p = 0.75)
Age of first reproduction	β = −0.47 (t_195_ = −2.01, p = 0.04)	β = 0.03 (t_195_ = 0.23, p = 0.82)
Number of offspring	β = 0.29 (t_195_ = 1.25, p = 0.21)	β = 0.06 (t_195_ = 0.42, p = 0.68)
Age of last reproduction	β = −0.13 (t_195_ = −0.54, p = 0.59)	β = 0.09 (t_195_ = 0.62, p = 0.53)
Age at menarche	β = −0.10 (t_195_ = −0.39, p = 0.69)	β = 0.12 (t_195_ = 0.83, p = 0.41)
Proportion of sons	β = 0.43 (t_195_ = 1.77, p = 0.08)	β = −0.07 (t_195_ = −0.47, p = 0.64)
Offspring survival	β = −0.15 (t_195_ = −0.65, p = 0.52)	β = 0.01 (t_195_ = 0.11, p = 0.91)

Tests were obtained from the multivariate regression model. Tests are provided for associations with hand asymmetry using all traits and only traits showing no directional asymmetry (DA). The multivariate F-test was not significant for both analyses (for all traits: F_7,195_ = 1.20, p = 0.30; for traits without DA: F_7,195_ = 0.23, p = 0.98).

## Discussion

Our results do not support findings from earlier studies. We did not find associations between hand FA and key female life history traits like age at first reproduction, age at menarche and number of children. If any, we found a trend opposite to what is expected for age at first reproduction, where women with more asymmetric hands started reproduction earlier.

There are several possible reasons for these results, although it remains difficult to pinpoint the most likely explanation. Firstly, the lack of significant associations may reflect a type II error. This possibility cannot be dismissed because the amount of variation in DI observed here was relatively low (i.e., average hypothetical repeatability of 0.07) and the total sample sizes only modest. However, compared to the median sample size of 100 in the human literature in this area [2], our sample size stands out. Indeed, about 75% of all studies in humans have sample sizes of 200 or smaller. However, associations, albeit not or only marginally significant, were in the opposite direction than observed earlier [6], [8]. If any, one would at least expect patterns to be in the direction predicted, making the likelihood of a type II error relatively small. Second, we have underestimated the degree of measurement error, because no repeated scans were available. However, between-scan variation in FA measurements has been shown to be low [15], suggesting that ME due to this cause is likely to be of minor importance. Third, associations between FA and life history may differ between traits used to measure FA. Earlier studies investigated breast FA [6], [8] and thus a trait with mainly soft tissues. In this study, the proportion of soft tissues in the hand is clearly much lower. Possibly, FA in soft tissues shows stronger associations with measures of quality, a hypothesis that remains yet to be tested. In addition, breast are sexually dimorphic and play a direct role in reproduction in women, which could result in stronger associations between breast FA and life history compared to other traits like hands. Nevertheless, it is also important to mention in this context that, albeit to a smaller extent, hands are also sexually dimorphic since the relative length of several digits differ between males and females [22]. Fourth, there may be direct effects of asymmetries. A recent review showed that attractiveness of body and face may require that the degree of asymmetry is visible during the evaluation of attractiveness [23]. This view is in contrast to the more generally held belief that DI is an individual property and that DI correlates with morphological features other than FA, affecting attractiveness. As DI increases FA, associations between FA and attractiveness are expected even if the asymmetry cannot be observed directly. The fact that associations between FA and attractiveness are much weaker when the asymmetries are hidden when attractiveness is scored (e.g., facial attractiveness correlates much weaker with body FA than with facial FA in spite of the fact that body FA is based on more traits), does not support this mechanism and suggest a small contribution of DI [15], [23]. The preference for more symmetric faces and bodies might instead be attributed to sensory bias, yet, little evidence is found for that [23], [24]. Clearly, more research is needed in this area, but the possible contribution of direct effects of asymmetry may be (albeit speculatively) relevant to explain patterns in the associations between FA and life history in humans. As breasts, and possibly the degree of asymmetry can be directly observed, breast FA may have a direct effect on attractiveness and as such affect sexual behavior. Possibly, more symmetric individuals that are judged or rated to be more attractive may as a direct consequence of this start reproducing earlier and/or have more children. Under this scenario, it is not unexpected that [8] did find associations between breast FA and both number of children and age of first reproduction, but not with age of menarche. While age of menarche is likely to be unrelated to observed attractiveness, number of children and age of first reproduction might be. Clearly, we cannot formally test this hypothesis. Overall reviews [2], [23] suggested that more research is needed to distinguish between the role of DI and observable asymmetry in humans. Fifth, we cannot rule out the possible effects of reproduction on hand asymmetry, because hand FA was measured after the women had ended their reproductive career. Possibly, a cost of reproduction might induce an increase in hand asymmetry. The latter scenario would involve modification of hand asymmetry in adults. Finally, we observed directional asymmetry in several hand measures, where on average the right hand was larger compared to the left. Since about 90% of the human population is right handed, such directional asymmetries have been interpreted as evidence for environmentally induced asymmetries, which are unrelated to DI [14], [15]. Indeed, it is known that the dominant hand experiences different mechanic loads and develops larger [15], [25]. It would thus have been better to control statistically for the effect of individual hand preference directly instead of correcting for DA. Unfortunately, we do not have that information available. In the only study we know of where a correction for hand preference is applied, however, such a correction did not change the overall outcome [15].

Correlations in the signed FA show that the hand develops as an integrated trait. This has been reported before [15] and is not unexpected given that a complex and integrated self-regulating network forms the basis of the development of the hand that already starts during the early limb bud formation [26]. On top of that, differences in mechanic loads between hands are likely to influence different digits simultaneously adding to the correlations in the signed FA's. If relatively strong, effects of handedness invalidate hand FA as a measure of DI (unless appropriately corrected for, [15] and see above). If so, it is not unexpected that we did not find any association of hand FA with life history. It also calls for caution in using hand asymmetry (and limb asymmetry in general) as a measure of DI in humans.

Despite our failure to replicate previous findings, negative results are as important as positive ones, because a lower likelihood of being published results in publication bias and incorrect conclusions. This point is highlighted in a recent meta-analysis of the literature in humans that found indications of a substantial publication bias in the field of FA [2]. The lack of association between hand FA and female life history reported here thus generates two working hypotheses: First, hand asymmetry in general may not be very suited to measure DI in humans due to behavioral lateralization's [15]. Second, there may be a greater role of FA in soft tissues as measures of DI and life history characteristics, and traits under sexual selection may yield stronger associations, perhaps due to direct effects on the outcomes. Clearly, and as emphasized so often, more research will be needed to evaluate the importance of FA as a proxy of individual quality and as a predictor of female life histories.
